# Mechanisms of DNA Damage Recognition by UDG and PARP1 in the Nucleosome

**DOI:** 10.3390/biom15050649

**Published:** 2025-04-30

**Authors:** Safwen Ghediri, Parvathy A. P. Sarma, Vinnarasi Saravanan, Corinne Abbadie, Ralf Blossey, Fabrizio Cleri

**Affiliations:** 1 Université de Lille, Institut d’Electronique Microelectronique et Nanotechnologie (IEMN CNRS, UMR8520) and Département de Physique, F59652 Villeneuve d’Ascq, France; safwen.ghediri.etu@univ-lille.fr (S.G.); parvathy.ananthapadmanabhasarma.etu@univ-lille.fr (P.A.P.S.); 2 Université de Lille, Unité de Glycobiologie Structurale et Fonctionnelle (UGSF CNRS UMR8576), F59000 Lille, France; vinnarasisaravanan94@gmail.com (V.S.); ralf.blossey@univ-lille.fr (R.B.); 3 Université de Lille, CNRS UMR9020 and Inserm U1277-CANTHER-Cancer Heterogeneity, Plasticity and Resistance to Therapies, F59000 Lille, France; corinne.abbadie@cnrs.fr; 4 Laboratory for Integrated Micro Mechatronics, LIMMS CNRS IRL2820 and University of Tokyo, Komaba, Meguro-ku, Tokyo 153-8505, Japan

**Keywords:** DNA damage, DNA repair, Single-strand break, Uracil base excision, Nucleosome, Molecular dynamics

## Abstract

The DNA base-excision repair (BER) pathway shares the second part of its enzymatic chain with the single-strand break (SSB) repair pathway. BER is initiated by a glycosylase, such as UDG, while SSBR is initiated by the multifunctional enzyme PARP1. The very early steps in the identification of the DNA damage are crucial to the correct initiation of the repair chains, and become even more complex when considering the realistic environment of damage to the DNA in the nucleosome. We performed molecular dynamics computer simulations of the interaction between the glycosylase UDG and a mutated uracil (as could result from oxidative deamination of cytosine), and between the Zn1-Zn2 fragment of PARP1 and a simulated SSB. The model system is a whole nucleosome in which DNA damage is inserted at various typical positions along the 145-bp sequence. It is shown that damage recognition by the enzymes requires very strict conditions, unlikely to be matched by pure random search along the DNA. We propose that mechanical deformation of the DNA around the defective sites may help signaling the presence of the defect, accelerating the search process.

## 1. Introduction

Free-radical attack can oxidize the nitrogenous bases within the DNA double helix, producing such typical species as oxidized guanine (known as 8-oxoG), or deaminated cytosine (which turns to uracil, U). Such defects are highly dangerous, since after replication they can originate inheritable mutations in the DNA; notably, both the 8-oxoG·C and the U·C pairs will produce a C·G→A·T mutation [1]. Instead, nucleophilic radical attack to the sugar-moiety of DNA, mainly leads to the formation of abasic sites and strand breaks (cuts of the phosphate backbone). Abasic sites occur when the N-glycosidic linkage between the sugar and the nitrogenous base is disrupted, resulting in the lack of base while the backbone remains intact [2]. On the other hand, strand breaks can occur due to the transfer of free radicals along the DNA molecule, either in the presence or absence of oxygen, where they attack typically the C5^′^ and C4^′^ carbon atom sites [2]. The strand break can occur on one, or both sides of the double helix (single- or double-strand break, respectively SSB or DSB). In the presence of oxygen, the result is the formation of “clean” breaks, terminated with 3^′^-OH and 5^′^-phosphate, and less likely with 3^′^-phosphate and 5^′^-OH. However, it has also been observed that attack on the C5^′^ or C4^′^ carbon can give rise, respectively, to 5^′^-deoxyribophosphate (dRP), $5^{\prime }$-aldehydes, or $3^{\prime }$-phosphoglycolate (PPG), or other more chemically complex (“dirty”) ends [3,4]. It is worth noting that dirty ends are virtually unprocessable, since DNA-ligases cannot reseal a SSB or a DSB unless the ends are clean [5]. For example, tyrosyl-DNA phosphodiesterase requires several hours to remove PPG from 3’ DNA termini [2,6].

**Figure 1:**
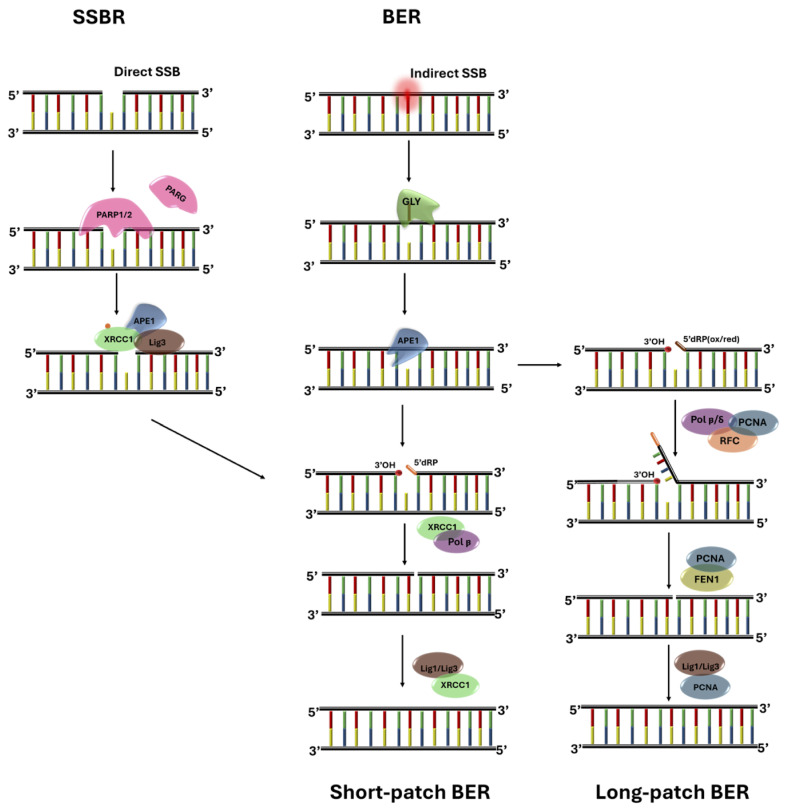
Model for BER and SSBR pathways. Base-excision repair (BER) is initiated by a glycosylase (GLY, in this work a UDG) that identifies and removes the wrong base, thus creating an abasic site; most abasic sites are processed by APE1, which cleaves the site and recruits Pol*β*. Pol*β* inserts the new nucleotide and repairs the 5^′^-deoxyribose phosphate (dRP) left by APE1: this creates a clean single-strand break (SSB). SSB-repair (SSBR) is initiated by PARP1, which (after a sequence of enzymatic reactions) serves as platform to recruit APE1, XRCC1 and other proteins; at this point the two pathways join, and can proceed to completion through either short- or long-patch BER.

Irrespective of its origin, DNA damage triggers specific chains of multi-enzyme reactions, globally known as DNA damage repair (DDR) pathways, which protect against genomic instability and accumulation of mutations. As a result, cells have developed multiple complex DNA repair pathways. There are no less than seven different known variants, which enable cells to identify the different types of damage, and repair DNA to the correct sequence as much as possible, in general with a very high success rate [7,8]. Among the different pathways, base excision repair (BER) and single-strand break repair (SSBR), which respectively address damage to individual bases and single-strand breaks, stand out because they share a large part of the complex physical-chemical steps, albeit starting from different premises ([9,10,11], Figure 1). For the above reasons, in this work we will look at BER/SSBR and, more specifically, at the two very early steps in this repair pathway by which two specific enzymes, uracil-DNA-glycosylase (UDG) and poly-ADP-ribose-polymerase-1 (PARP1), identify the initial molecular conformation of, respectively, deaminated-cytosine (that is, uracil), and a single-strand break (SSB).

The very first step of any attempt to repair a damage highly localized to one specific position on the DNA, most often to just one base, is a formidable task: finding this site amid billions of bases of undamaged DNA. Pure 3D-diffusion of proteins, randomly searching DNA stretches for a possible damage has long been thought to be insufficient for the DNA-repair process. Repair proteins must be extremely efficient to inspect all bases in the genome (∼3$\times 10^{9}$ base pairs), and detect and repair damaged bases (around 1 in $10^{6}$ bases per cell daily). Given that for each repair protein in the pathway there could be ∼10^3^–10^4^ copies in the nucleus, each such protein (excluding redundancy) should be able to sample around 100,000 base pairs of DNA in every cell cycle (about 12 to 24 h [12]). The fact that, in practice, repair proteins can achieve their job much faster, largely defying even “facilitated diffusion” models [13,14,15], points to the existence of better than purely stochastic search mechanisms. Among the possible solutions, we suggested [16,17,18] that local deformation at the defect sites could make the damage more “visible”, by inducing a mechanical stress field along the DNA backbone that facilitates target identification by the repair proteins.

Experiments giving access to the molecular details of protein-DNA interaction (such as X-ray diffraction, nuclear-magnetic resonance, electron cryo-microscopy) are quite difficult, and almost invariably involve a fragment of the protein (more rarely the entire protein unless it is relatively small, e.g., in the range of 2–300 amino acids) and a very short DNA oligomer. By contrast, DNA in the chromatin is typically packaged as “beads-on-a-string” into nucleosomes, clusters of eight histone proteins about which a stretch of ∼50 nm of DNA (147 base-pairs) is continuously wound into a roughly spherical object (the nucleosome core particle, NCP) of about 11 nm diameter; pairs of nucleosomes are connected by stretches of “linker” DNA with length variable between some 20 to about 200 base-pairs (that is, about ∼5 to ∼50 nm).

Therefore, the free-DNA short oligomers typically used in experiments could only be a rather poor representation of the actual DNA in the chromatin; even the (more or less) long DNA linkers could not be represented as free floating fragments, but are entropically and mechanically constrained by their ends attached to each pair of nucleosomes. On the other hand, studying the protein-DNA interactions in the more realistic context of the nucleosome and NCP is a far more complex task, and the examples are consequently more sparse: actually, out of more than 230,000 molecular structures currently available from the RCSB Protein Data Base, less than 300 report a nucleosome-protein complex. Moreover, since on average about 75% of the DNA is wrapped into nucleosomes, the external damage such as radiation-induced should be more often localized at nucleosomes. Given such a context, molecular computer simulation could be of great help to shed light on the detailed mechanisms of action of the many physical-chemical steps involved in DNA repair in such a complex supramolecular structure.

In this work we explore by means of molecular dynamics (MD) computer simulations, the DNA-protein interactions involving UDG and PARP1 enzymes, in the full environment of an isolated nucleosome (actually a NCP, i.e., without linkers). We manually introduce constructs of DNA damage at various sites around the nucleosome, in the form (1) of a uracil base, targeted by UDG; and (2) of a clean single-strand break, targeted by PARP1. Given the structural differences between the two defects, the molecular mechanism by which each enzyme identifies the defect are very different. However, in both cases there are similar basic questions underlying, namely, whether the enzyme actively modifies the defect site during the recognition, or rather the defect site “exposes” itself to facilitate the identification. For UDG to target the uracil, the wrong base must be flipped out of the DNA axis in extrahelical position, such that the UDG binding pocket can wrap around it and proceed to the excision; for PARP1 to target the SSB, the two ends of the cut DNA must open-up and twist, so that the two zinc fingers of PARP1 can bind at each end, and maintain the break wide open for the repair chain to proceed. In both cases, depending on the ordering of the elementary steps of the process, such an interaction could represent either a “bind-then-bend” (the enzyme actively deforms the defect site after finding it), or rather a “bend-then-bind” (the defect is spontaneously modified prior to binding) sequence [19,20,21,22,23,24]. This issue becomes further complicated when DNA is wrapped around the nucleosome, because of the steric hindrance and extra charges provided by the histone proteins, and notably the unwieldy histone tails. As a matter of fact, very few experiments are available to date for UDG interacting with a nucleosome, e.g., [25,26,27,28,29], and PARP1 interacting with nucleosomal DNA [30,31], none of which provided detailed molecular structures of the full protein-nucleosome complex. This underscores the importance and opportunity of addressing these issues also by computer simulation, at least to provide a first glance about the numerous open questions.

We already addressed in two recent works the computational study of both these enzyme-DNA complex. For UDG [32], we started by looking at the detailed energetics and kinetics of extrahelical base-flipping in a short DNA oligomer, by performing free-energy calculations with a metadynamics approach. For PARP1 [18], we adopted the special structure of DNA hairpin with a missing nucleotide to imitate a SSB, already used in several experimental studies [33,34], and carried out extensive MD simulations to characterize the microscopic conditions and dynamics of damage identification by the Zn1-Zn2 zinc-finger fragments of the enzyme. With the present work, we extend our investigation to protein-DNA interactions in a whole nucleosome, with the aim of characterizing the early steps of damage identification in the BER/SSBR pathways in a realistic environment, possibly more relevant to the development of DNA damage repair within the nuclear chromatin.

## 2. Methods

To elucidate the molecular interactions in a model nucleosome between, respectively, UDG and a flipped-out uracil, and PARP1 and a SSB, we designed a complete simulation protocol, starting with (i) the design of the atomic structure of the nucleosome, DNA and protein fragments; then (ii) docking of the rigid fragments (protein to the DNA) with an approximate free-energy functional; followed by (iii) all-atom molecular dynamics (MD) simulations with a state-of-the-art empirical force field of the complete protein+nucleosome, embedded in a large box of water and ions for a physiologic solution, and (iv) detailed analysis of the molecular configurations and structural dynamics.

### 2.1. Docking

Docking of proteins and nucleic acids is still a rapidly evolving field of research. We tested different methods available as online server platforms: HADDOCK [35,36], HDOCK [37] and the very recent PyDockDNA [38]. For the PARP-SSB configurations only HADDOCK was capable of providing consistent results comparable to the available experimental information. Similar to our previous study [18], we used the PARP1 fragment including the two Zn-finger domains, and a DNA decamer extracted from one of the three sites chosen along the nucleosome (see below). It is worth noting that all experimental structural studies focussed the attention on the Zn1-Zn2 fragment, which appears to be the actual damage-sensing domain of PARP, while the Zn3 and its other domains, the BRCT auto-modification domain, WGR nucleic-acid binding domain, and the catalytic C-terminal domain, are needed only for the downstream functions of PARP. Hence, including the full-scale PARP1 structure would not add much to the study, as far as SSB detection is concerned, but rather add just some further steric constraints on the docking. A SSB was introduced midway in the 10mer by removing one full nucleotide, following the protocol established in [18]. As a starting point, the base-stacking loop in Zn2 was always designated as the active residue for interaction with the $3^{\prime }$ end of the SSB. HADDOCK bootstrap parameters were chosen such that we could typically aim at the regions including the “trademark” interactions between Zn1-Zn2 and the SSB [33]. Best-fit clusters of candidate high-scoring poses were taken from the docking process. After the docking, the selected complex PARP1+10mer was put back into the nucleosome with the Chimera 1.17 software [39], to be used as the initial configuration of subsequent MD simulations.

For UDG, after testing different methods among the available online platforms, PyDockDNA turned out to be the only one capable of providing reliable results. We started the docking using the molecular structure of UDG from RCSB Protein Database entry 1EMH, and a DNA-X generic fragment of seven base-pairs including a flipped uracil or thymine, from our previous study; for each of the six interaction sites along the nucleosome (see below), we replaced the DNA-X sequence with the local nucleosomal 7-mer sequence, however without allowing for structural relaxation. To enhance accuracy, we incorporated the position of the UDG pocket interacting with the flipped nucleotide into the docking parameters. PyDockDNA generated the top 100 docking results, from which we selected the most suitable candidate, based on a comparison (minimum RMSD) of the flipped-nt geometric positioning in the pocket with respect to the experimental structures 1SSP and 1EMH. Then, the selected UDG+7mer complex was repositioned back in its nucleosome site, to start the MD equilibration.

### 2.2. Molecular Dynamics

All the MD simulations and most data analysis, were performed with the GROMACS 2020.4 package [40,41]. The Amber14SB force-field was used in the simulations [42], with extra parameters for Zn ion interaction from Macchiagodena et al. [43]; notably, Amber14 already includes the latest Parmbsc1 extension for nucleic acids [44]. We note that the Amber14SB is the optimal choice for working with the TIP3P water model; the most recent Amber19SB could be a somewhat improved solution, however it requires to work with the OPC water model that, albeit more accurate, is also much more expensive (about 33% more computational time w/r to TIP3P). Given the very large size of the molecular systems that we use, the simpler and widespread TIP3P water model still remains a good compromise. Concerning DNA, OL15 has the same level of performance as PARMbsc1 [45].

To ensure the primary stability of the post-docked configurations, we first performed energy-minimization using a steepest-descent algorithm, followed by {NVT}-{NPT} equilibration for 150 ps, to bring the systems at 310 K and 1 bar pressure. The ensembles of DNA-proteins were solvated in a water box of size 15 × 15 × 15 nm^3^, with about 106,500 water molecules, along with Na^+^ and Cl^−^ ions for neutralizing the system charge and maintaining a physiological 0.1 M salt concentration. In some instances we also performed rapid annealing and quenching cycles, to improve the mutual positions of protein and DNA, for example after a manual adjustment necessary to remove a steric clash. Each cycle was typically performed by ramping up the temperature from 310 K to 400 K, and then back to 310 K, in steps of 10 K for 500 ps each. Usually, during the annealing cycle the DNA was frozen in its conformation and only the protein was left free to adjust.

Coulomb forces were summed with particle-mesh Ewald sum (PME), using a real-space cutoff of 1.2 nm, equal to the cut-off radius of shifted Van der Waals potentials. We used rigid bonds for the water molecules, which allowed integration of the equations of motion with a time step of 2 fs for the thermal equilibration phases, and 1 or 2 fs for production runs.

Trajectory clustering analysis was performed using a special subprogram of GROMACS. The MD simulation stores ’frames’ containing the positions, velocities and forces of all particles in the system, at prescribed intervals (typically every 10 to 50 ps, or longer: a 1 µs-long MD run can store as much as 100,000 frames of about 2 Mbytes each, resulting in data files with size of hundreds of Gbytes); the subprogram calculates a matrix of root-mean-squared displacements (RMSD) between each pair of frames, by comparing the positions of each atom in the pair; then, RMSD values are grouped according to a cut-off criterion, and clusters of similar frames, typically separated by a small enough RMSD, are detected. The cluster(s) with the highest number of members, and those which are more frequently sampled over the entire simulation time, provide an indication of the ’best-average’ molecular configurations, thus allowing to bypass the noise of the short-time atomic fluctuations.

Free energy analysis and contact surface calculations were estimated by the PDBePisa web-based utility [46], by extracting a few selected configurations in PDB format from the MD trajectory. The resulting values are approximated, however they can provide a qualitative appraisal of the binding energies and chemical affinity involved in the protein-DNA interaction.

### 2.3. Nucleosome

**Figure 2:**
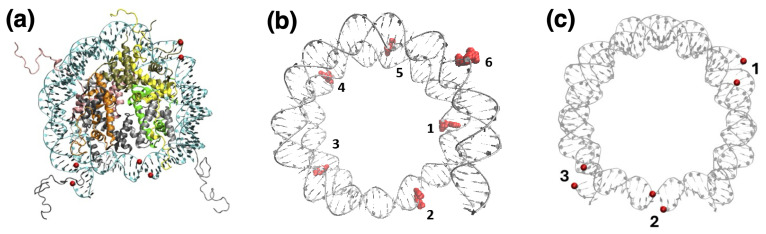
(**a**) Top view of the model nucleosome; histone proteins represented as color ribbons; DNA in cyan. (**b**) UDG/DNA interaction sites, with the positions of the U/T substitutions indicated by VdW spheres, and numbered 1 = SHL+1.5, 2 = SHL−0 (dyad), 3 = SHL+6.5, 4 = SHL+4.5, 5 = SHL+3, 6 = SHL−5. (**c**) PARP1/DNA interaction sites, with the positions of the single-strand break numbered 1 = SHL+5, 2 = SHL−0 (dyad), 3 = SHL−0.5; the free SSB ends are represented by colored spheres.

The initial molecular configuration of the nucleosome is taken from the RSCB Protein Database, entry 7OHC [47]. This is a cryo-microscpy structure of the entire histone octamer with 145 DNA base-pairs resolved at an average RMS of 2.5 Å, reconstituted from human nuclear extract expressed in *E. coli*; the missing histone residues were completed with the Swiss-Model utility of SwissProt [48] to obtain a complete nucleosome including the full-length histone tails. The 145-bp DNA has the Widom 601-sequence [49], originally selected to maximize the binding affinity to the histone octamer; to obtain a model structure useful for our computer simulations, we removed all the crystallization water molecules. DNA in the nucleosome is wrapped left-handed about the histone core, making two nearly complete turns that join at the dyad symmetry point; the two DNA turns define two circles lying in two ideally parallel planes, with a superhelical-symmetry axis (SHL) perpendicular to the center of the circles; the relaxed DNA double helix makes a complete twist around its double-helical axis, about every 10.4 bp; correspondingly, 14 contact points between DNA and the histone core proteins can be identified within the nucleosome structure, loosely situated at the minor groove locations facing inwards. Based on these standard geometrical features, we defined the sites where to place DNA damage in the form of either a uracil (oxidized cytosine), or a single-strand break (SSB), in structurally significant positions (see below). Figure 2 provides a summary of the nucleosome structure and damage locations in the two cases. For UDG, we selected: two sites (4 and 5) that are more easily accessible, but differ in the proximity of the H2A histone tail for the second one, and none for the first; then, two sites (1 and 3) that are similarly exposed but differ in being on either side of the H4 histone tail; a “dyad” site (the 2) that is close to the previous 5, but it is more openly accessible; and two sites (5 and 6) that are less easily accessible, and have part of the UDG protein sterically limited by the nucleosome contact, but in a loop far from the binding pocket. For PARP1, the more restricted choice (simulation time was longer) was limited to three positions: an “average” one, somewhere midway along the DNA gyre (the n.1); one close to the entry branch of the nucleosome (n.3); and one in the (apparently) easily accessible dyad (n.2) where only one dsDNA loop is found.

## 3. Results: Uracil and UDG

### 3.1. Structures of UDG and Flipped-Out Nucleotides

The uracil defective nucleotide is initially arranged in a fully-extrahelical position, based on the findings of our metadynamics study on short DNA oligomers [32], i.e., rotated at 180° about the O-P-O phosphate chain with respect to its equilibrium position. This conformation may equally represent the uracil, either spontaneously fluctuating before the interaction with UDG (in a “bend-then-bind” sequence [19,20]), or right after UDG could perform a pinch-and-pull mechanical action (in a “bind-then-bend” sequence [50,51]). To assess the physical-chemical selectivity of UDG towards the mutated vs. non-mutated sequence, we also ran MD simulations on identical configurations, by replacing U with T, in a correct T·A base-pair.

For the molecular structure of the human UDG glycosylase, we started from two experimental structures of the enzyme interacting with a short DNA oligomer, available in the RCSB PDB archive, the 1EMH and the 1SSP; the two are very similar, in that they both include a wild-type UDG bound to a DNA decamer containing in the 5th base, respectively, an uncleaved uracil [52], or a cleaved uracil [53]. It is worth noting that the two molecular structures are nearly identical, with the cleaved U displaced by just 0.12 nm inside the UDG pocket after the cut.

As said, six distinct configurations of the nucleosome structure were generated, each featuring two variations: one with thymine (T) and the other with uracil (U). The substitution of T with U was performed using the Chimera *swapna* utility [39]. The selection of specific positions for these modifications was guided by geometrical criteria. The level of accessibility was defined based on two factors: (i) the solvent exposure of the nucleotide, i.e., whether it is oriented toward the interior of the nucleosome (hard), thereby restricting UDG access; or facing outwards (easy), where it remains fully exposed; or intermediate between the two (medium); and (ii) the eventual proximity of a histone tail, that may perturb UDG while interacting with the flipped nucleotide. This criterion allowed for a systematic evaluation of how nucleosomal positioning influences the recognition and excision of uracil by UDG.

### 3.2. Nucleosome Accessibility and UDG Positioning

Compared to the ’clean’ experiments with short DNA oligomers, the nucleosome presents UDG with a much more rugged landscape: the DNA is bent with a tight curvature; the major/minor groove widths, base-pair and stacking parameters are fluctuating to rather different values from those of the ideal, straight B-DNA; histones are partially hiding access to DNA, and their long, positively-charged tails fluctuate around the structure, representing further hindrance. Therefore, comparing the set of six different sites of interaction between UDG and a flipped nucleotide along the nucleosome, allows to understand at least some necessary conditions for a correct binding and subsequent excision action of the enzyme (that is, an arrangement of the complex comparable to the experiments, e.g., [52,53,54,55], which typically depict the final state of the DNA-enzyme interaction).

The initial docking chiefly aimed at positioning the flipped nucleotide in the correct position inside the binding pocket. However, this microscopic configuration can be equally achieved with many different macroscopic arrangements of the whole UDG about the DNA. A correct arrangement should allow at least to: (i) achieve a proper orientation of Leu272, which should enter the DNA minor groove close to the phosphate of the flipped-out nucleotide, and sandwiched between its two neighboring $5^{\prime }/3^{\prime }$ nucleotides; and (ii) to place the 146–149 and 211–216 loops of UDG facing the major groove, next to the flipped nucleotide. Notably, in order to preserve enough space for the central nucleotide to flip-out through the major groove, these two loops must keep a distance of at least 1.5 nm from the Leu272 loop. It will be seen that the geometrical constraints of the nucleosome structure do not always permit such ideal conformations of UDG about the DNA, thereby motivating the experimentally-observed rotational dependence (weakening) of UDG catalytic activity in the nucleosome [25,26,27,56].

The flipped-out nucleotide must fit inside the binding pocket, right next to the main beta-sheet, where it should get bound by H-bonds with the two key residues Asn204 and Phe158. If the base is uracil, then Gln144 helped by His268 will make the nucleophilic attack that cuts the glycosidic bond; otherwise the base will be ignored. (In practice, UDG may also excise a correct pyrimidine, however the rate of such errors is in the range $10^{-8}$ [12]). Note that given the size of the pocket, purines cannot fit in. As it will be seen, although all the initially-docked complexes display a correct positioning of the flipped nucleotide in the binding pocket, the subsequent dynamics shows that the interaction can only be maintained when the geometrical constraints on UDG-DNA steric arrangement are satisfied.

Notably, the UDG positioning can also display a variable degree of sequence-dependence, since the extrahelical flipping induces a substantial phosphate backbone deformation, at least of the base-pairs immediately adjacent the $5^{\prime }$ and $3^{\prime }$ sides, the DNA getting kinked at the flipped site. Our chosen interaction sites along the nucleosome have quite different flanking tetramer sequences; Table 1 provides an estimate of the corresponding flexibility (marked by an increasing number of ’*’), defined as average of stretching, bending and twisting constants [57]. Given the important deformations of the DNA backbone observed, such different elastic properties should impact on the relative ease of UDG positioning during its search for defects along the nucleosome.

### 3.3. Structural Dynamics of UDG Interactions in the Nucleosome

Table 1 summarizes a number of results for the 6+6 UDG-DNA interaction sites calculated with the PDBePisa utility [46], by averaging over frames extracted from the last 10 ns of each MD trajectory. Each row corresponds to a substitution in the DNA chain/nucleotide indicated in column 6 (for example, the first row is a flipped-out thymine in chain I, position 16 (corresponding to SHL+1.5), the second row is a uracil in the same position, and so on). Whenever two values are shown separated by a “/”, the first refers to the total, and the second to the interaction with only the flipped nucleotide. $\Delta G_{S}$ in column 11 indicates the solvation free-energy gain upon formation of the interface, in kcal/mol (not a *total* free-energy of the compound formation). The value is calculated as difference in solvation energies between the isolated and interfacing structures: a negative $\Delta G_{S}$ corresponds to hydrophobic interfaces, or positive protein affinity. Note that this value does not include the effect of satisfied hydrogen bonds and salt bridges across the interface given in columns 8–10; the average error on each calculated frame is between 10–20%, however when averaged over the last 10-ns the uncertainty on the value reported in the table is about 10%. The interface area in column 12 is calculated as the difference in total solvent-accessible surface areas (Å^2^), between the isolated and interfacing structures, and divided by 2.

In general, it may be noted that both the solvation energy and the interface area are systematically larger for the U vs. T substitutions. Also, looking at the fraction of H-bonds, the ratio to the total is always favorable to the U vs. T. However, by looking at the overall geometry after the 100-ns dynamics, we find that only the configurations SHL−0 (or I-3), SHL+3 ( I-34) and SHL+4.5 (I-46) still display a correct, or near-correct binding. We will discuss below two typical examples of “easy” and “hard” positions.

**Figure 3:**
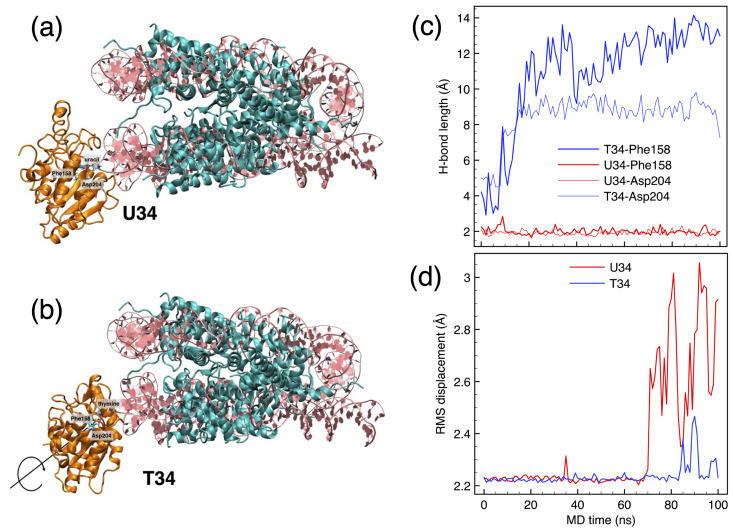
(**a**,**b**) Best cluster of UDG at nucleosome position SHL+3, interacting with U34 and T34 nucleotides, after 100 ns of MD. DNA is depicted in pink ribbons, UDG in orange ribbons, histones in cyan ribbons. (**c**) Time evolution of the H-bonds initially established between the O4 atoms in the flipped out U/T (red/blue plots, respectively) with the N in Phe158 and N*δ* in Asn2044. (**d**) Root-mean-squared displacement of UDG interacting with U34 and T34, compared to the experimental 1EMH structure.

For position SHL+3, both T34 and U34 start from a quite correct UDG/DNA docking arrangement, Leu272 is intercalated in the minor groove between the flanking nucleotides 33–35, and H-bonds are formed between the flipped base and the Phe158 and Asn204. However, very soon in the dynamics the H-bonds are broken in the T34 (blue plots in Figure 3c), which eventually is expelled from the binding pocket; at the same time, Leu272 is pushed out of the minor groove, and UDG makes a rigid rotation about its original arrangement on the DNA (Figure 3b). By contrast, U34 retains the correct binding and interaction configuration for the whole duration of the dynamical trajectory (see Figure 3a and red plots in Figure 3c). It could be interesting to note that, by looking at the average RMSD along the trajectory (Figure 3d), U34 shows larger differences than T34, when compared to the reference experimental structure 1EMH; the partial detachment of UDG in the case of T34 leaves the overall structure of the protein more compact, whereas some rearrangement to accommodate U34 is necessary, however mostly concentrated on some side chains. A very similar response is observed when looking at position SHL+4.5, in which case the U46 remains correctly bound inside the binding pocket of UDG for the whole duration of the 100-ns MD trajectory, while T46 is rapidly pushed out of the pocket.

**Figure 4:**
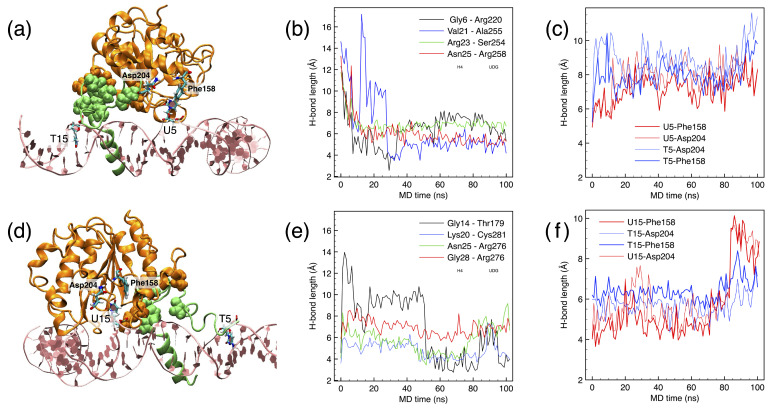
(**a**) Best cluster for UDG at uracil in position U5 (SHL+0.5). UDG in orange ribbons and histone H4 in green ribbons; the contacts between UDG and H4 are highlighted by VdW spheres; the site T15 is also shown for reference. (**b**) Time evolution of the contacts, represented by the distance between the centers of mass of the respective amino acids. (**c**) H-bonds between the O4 atom in the flipped out U/T (red/blue plots, respectively) with the N in Phe158 and N*δ* in Asn204. (**d**–**f**) Same as (**a**–**c**) for UDG at uracil in position U15 (SHL+1.5), same color codes.

By contrast, positions SHL+0.5 and +1.5 are typical examples of difficult positions for fitting UDG (“hard” in Table 1 above), the flipped nucleotide being at the last, barely accessible position next to the histone core; moreover, the N-terminal of histone H4 runs through the minor groove close to the target DNA site (see Figure 4a,d). The site U5/T5 (facing “above” the nucleosome) is right next to the H4 histone (Figure 4a), whose contact with UDG forces a non-correct positioning at the DNA backbone. As a result, while Leu272 tries to keep the intercalation in the minor groove, the flipped-out nucleotide cannot properly accommodate in the binding pocket, as shown by the large distance maintained between the O4 atom of either U5 or T5, and the trademark H-bonding N and N*δ* atoms of Phe158 and Asn204 (Figure 4b). Moreover, the flipped nucleotide is here flanked by quite rigid tetramers (Table 1), which contribute to a more difficult accommodation. Being one full double-helix turn away from the previous SHL+0.5, UDG at SHL+1.5 is now found on the opposite side of the same H4 histone (compare Figure 4a and Figure 4d). The histone tail interacts with the side chains of UDG (see Figure 4e), forcing a non-ideal conformation of the enzyme. Also in this case, like for SHL+0.5, the distance between the flipped nucleotide and the key residues 158/204 in the binding pocket clearly shows the incoherent adaptation of UDG on the target base, which is progressively pushed out of the pocket (see the steep increase of all plots at time t∼80 ns in Figure 4f).

At rotational positions such as SHL–5 and +6.5, a specific difficulty of UDG interacting with DNA in the nucleosome is highlighted. In this case, the side-loop 114–116 of UDG correctly docked on the flipped-out nucleotide on one nucleosome turn, gets in contact with the DNA in the opposite nucleosome turn. Such a contact establishes several dynamical H-bonds and electrostatic contacts, which force the two UDG loops 146–149 and 212–215 deeper inside the DNA major groove. As a result, both T and U remain inside the binding pocket after the initial docking; however, the deformation imposed to UDG would make it difficult for the target nucleotide to flip-out, in the first place, because of the numerous steric clashes it would face in its rotational movement across the major groove.

These results point to the fact that, when considering DNA damage in the nucleosome, an enzyme like UDG must satisfy a number of constraints before finding a “correct” arrangement on the DNA backbone. The molecular structure must adjust on the target site in order to form the search-complex, and subsequently promote the interrogation-complex. At this stage it is unclear whether enzyme-assisted flipping of the target base is the most efficient way to proceed to the interrogation-complex, or rather the spontaneous rotation of the bases by thermal fluctuation and mechanical forces may be the preferable route. If we take the examples shown in this section as a representative sample of the different positions around the nucleosome, it turns out that only ∼25% of the bases around the nucleosome could be readily accessible, while the rest is either inaccessible, or is perturbed to a different degree by the nucleosome structure, notably the histone tails.

## 4. Results: Single-Strand Breaks and PARP1

### 4.1. Structures of PARP1 and Single-Strand Breaks

The few experimental studies of the interaction of PARP1 with isolated mononucleosome particles have repeatedly shown PARP1 bound to the blunt ends of DNA that are found at the entry/exit of the nucleosome [30,31,58]. Recent studies also demonstrated that two or more molecules of PARP1 can bind to both nucleosome free ends, mimicking (one half of) DNA double-strand break (DSB) damage [31]. The binding of PARP1 to the nucleosome brings about allosteric changes not just in the enzyme itself, but it also may partially unfold the nucleosomal DNA, by reducing the mobility at certain regions [30]. This could also be possibly due to destabilization of the intra-nucleosome interactions among the core histones [30]. Thus, it is surmised that the interaction of PARP1 with nucleosomes could drive some form of reorganization of the nucleosome itself, and the magnitude of local changes depends upon the strength (and number) of PARP1 attachment(s). It was indeed observed that multiple molecules of PARP1 bring about extensive rearrangement and unfolding of the nucleosome particle, while a single copy of PARP can only bring about local changes at the binding site [31]. In certain cases, PARP1 binding kinetics is accelerated by the presence of a H2A.X histone variant, and may therefore contribute to PARP1 accumulation, chromatin reorganization, and PAR synthesis [59]. Moreover, the study by Luger et al. [58] showed a large reduction of the binding affinity for nucleosomes lacking the linker-DNA fragment (i.e., the nucleosome core particle, NCP). Given the above experimental landscape, it is however worth reminding that there are no studies that focused on the molecular details of PARP1 interacting at the site of a SSB located on the body of nucleosomal DNA. Such a situation provides a critical motivation for our investigation by molecular simulations of the interaction of PARP1 with DNA damage directly in the nucleosome body, and in particular the role of SSB that is traditionally much less studied than the more dangerous DSB.

Starting from the results of our previous work on the free DNA hairpin [18], the structure of the Zn1-Zn2 finger domain of PARP1 was taken from the experimental NMR data deposited in RCSB under the entry 2N8A [33]. For the sake of comparison, we also took the corresponding fragment from the AlphaFold2 repository [60,61], entry P09874. The structure obtained from 2N8A consists mainly of the Zn1-Zn2 fragment of PARP1 (residues 1–201). AlphaFold2 instead predicted the entire structure of PARP1, with all its domains: in the AlphaFold2 model, all major domains of PARP1 are comparable to the corresponding solution structure obtained from NMR studies within a reasonable confidence interval, except the disordered linker stretch of 13 amino acids connecting the Zn1 and Zn2 domains.

**Figure 5:**
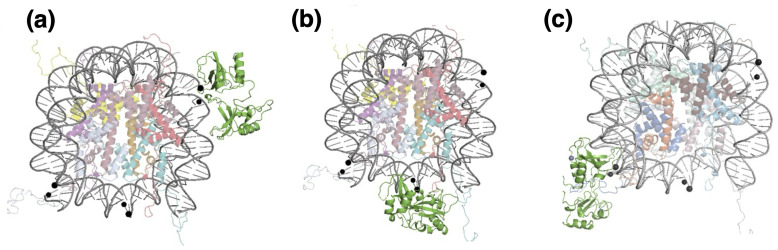
The initial configurations of the three SSB positions around the model nucleosome with the docked PARP1 (Zn1-Zn2 represented in green ribbons). (**a**–**c**) respectively depicting SSB site 1, 2 (dyad) and 3.

Single-strand breaks (SSB) were simulated by removing a whole nucleotide, and fixing the hanging DNA termini by $3^{\prime }$-OH and $5^{\prime }$-dRP ends; as a consequence, the nucleotide in the opposite strand remains unpaired. Note that to maintain the similarity with both our previous study [18] and the experimental set up [33], the $5^{\prime }$ is terminated with a dRP instead of a phosphate, since PARP1 apparently displays a greater binding affinity to the “dirty” ends created by the strand breaks; for the same reason, we adjusted with the Chimera *swapna* utility the local sequence of nucleotides flanking the SSB to be always guanines, i.e., the local dsDNA sequence around the missing nucleotide ’*’ is always $\left(\begin{array}{c} 5^{\prime }-G*G-3^{\prime }\\ 3^{\prime }-CTC-5^{\prime } \end{array}\right)$. Figure 5 depicts the nucleosome containing three SSBs at distinct positions: site 1 is within the nucleosome, at superhelical position -5 (SHL−5); site 2 is at the dyad, position SHL−0; and site 3 is just near the entry/exit of the nucleosome, SHL−7.

As an initial check prior to introducing the PARP1 interaction, we firstly tested the thermal and mechanical stability of the model nucleosome including the three SSBs. In particular, we wanted to verify whether there could be observed a spontaneous “opening” of the SSB free ends just by thermal fluctuations, and any spontaneous mechanical deformation near the SSB site, without the help of PARP1 [18,33]. For this purpose, a first series of 200-ns MD simulations were run for the nucleosome system with the three SSB and no PARP1. Note that all the SSBs are far apart from each other and lie in same DNA strand, so that they cannot interact mechanically, nor evolve into a kind of DSB even if at a long distance.

**Figure 6:**
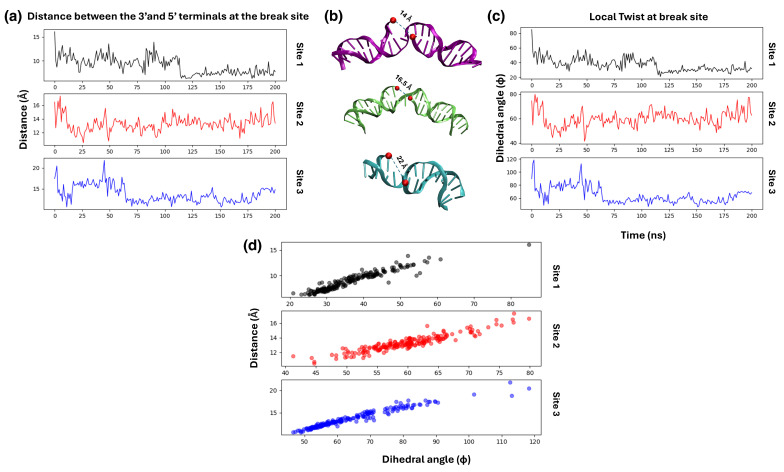
(**a**) Gap opening distance between the $3^{\prime }$ and $5^{\prime }$ ends of the each SSB break site in the free nucleosome (without any PARP1). (**b**) The corresponding snapshots of each SSB at the maximum opening. (**c**) Evolution of local twist Φ at each SSB site over 200 ns of molecular dynamics. (**d**) Correlation of the gap opening $d_{g}$ with the dihedral angle Φ.

The SSB gap distance $d_{g}$ is calculated between the instantaneous positions of the C3^′^ atoms in the 5^′^ and 3^′^ ends. After 200-ns of MD simulation, it is observed that the SSB opening between the free ends fluctuates between $d_{g}$ = 1.5 to 2 nm, as shown in the plot in Figure 6a. Upon comparing individual SSB sites, it is observed that site 1 and site 2 display very minimal fluctuations in the $d_{g}$ value, with site 1 closing back to *d_g_*∼0.8–1 nm in the second half of the trajectory, while site 3 (close to the entry point of the NCP) does open up to as much as 2.2 nm, due to thermal fluctuations. For comparison, in our previous work [18] the SSB in the hairpin-shaped DNA—but not in the long free-DNA—was observed to open up spontaneously due to thermal fluctuations, even in the absence of PARP1.

The next preliminary check is to test whether mechanical deformation in the SSB site affects the opening and closing of the gap. For this purpose, we calculated the local twist at the break site, defined as the value of the dihedral angle Φ between the C3^′^ atoms of the two base-pairs flanking either side of the unpaired nucleotide. Given that the DNA helix is twisted by about 34 degrees at each base-pair, the normal value of Φ should be about 60–70 degrees. Figure 6c,d respectively show the evolution of the local twist Φ over 200 ns, and the correlation between the gap opening distance $d_{g}$ and the angle Φ. The last plot (d) demonstrates that the local twist and the gap distance are linearly related, where an increase in the SSB gap opening is associated with a large local over-twisting of the double helix. For site 1, the closing of $d_{g}$ to ∼1 nm corresponds to a under-twisting down to Φ∼35–40 deg. The dyad site 2 displays a nearly unperturbed configuration, in both the $d_{g}$ and Φ values. Interestingly, the larger values of $d_{g}$ and Φ for site 3 compared to the other two sites should be due to its position at the entry of the nucleosome. This particular SSB position leads to the formation of a short stretch of 5-nucleotide dsDNA at the entry of nucleosome, right before the SSB, which is a highly unstable configuration quite prone to opening and deformation under thermal fluctuations.

### 4.2. Analysing the Zn-Finger Domain Contacts with SSB

SSB identification by PARP1 Zn-finger domains is predominantly characterized by a phosphate-backbone loop and base stacking-loop of the protein fragment, interacting with the strand break site [33]. The latter smFRET experimental study also showed that the Zn2 domain has a higher affinity to the damage site, compared to Zn1 [34]. While Zn2 identifies the break, and brings about conformational changes in the DNA, Zn1 would further add more interaction with the break, which eventually helps the allosteric changes in the whole PARP1 molecule. The major interaction occurs between Leu151 of Zn2 and the SSB $3^{\prime }$ end, which induce PARP1 to undergo some allosteric shift while strengthening its binding to the SSB. Zn1 then enhances these interactions, by adding further contact points at the SSB $5^{\prime }$ end mainly via its Phe44 residue, thus stabilizing the binding, to initiate PAR-chain synthesis and chromatin restructuring [33].

We attempted to replicate the specific contacts mentioned above, between PARP1 zinc-finger domains and the three SSB sites in nucleosomal DNA, similar to the protocol and observations already followed for the DNA-hairpin structure [18]. We designed the initial structure by docking three replicas of the Zn1-Zn2-finger domains to each of the SSB sites around the nucleosome, with the protocol discussed in the Materials section, using HADDOCK 2.4 and Chimera 1.17 public-domain softwares. We merged both these methods together due to the difficulty in achieving a good quality of the contacts between the Zn-finger domains and the SSB. In retrospect, this may be due to the limitations of HADDOCK in dealing with too complex and too large molecular assemblies. Also, we had already observed [18] that the “right” contacts are necessary for bringing about the subsequent opening and bending of the SSB. In fact, the docked clusters obtained from HADDOCK had some of the interactions observed in the experimental NMR structure for the DNA hairpin; from these clusters, the best docking pose were selected based on their RMSD values, as the initial configuration; then, Chimera was used to further improve the adjustment of the interacting moieties, and get the final structures as shown in Figure 5. These structures were then used as initial configurations for 200-ns MD simulations at 310 K and 1 atm.

**Figure 7:**
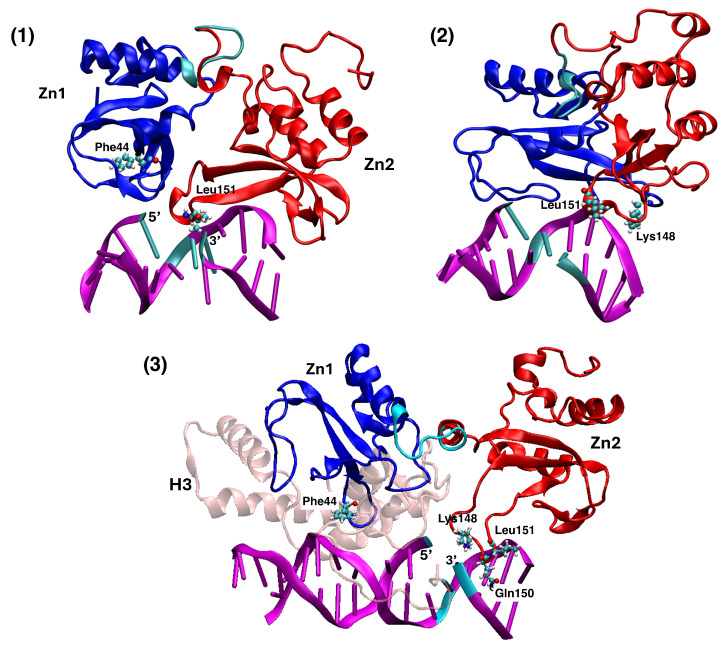
Configurations depicting the interaction of the Zn1 (blue ribbons) and Zn2 (red ribbons) with the DNA fragment including the SSB (purple), for the sites (1), (2) and (3). The best clusters represented are obtained from the 200-ns MD trajectory, by setting a RMSD cutoff of 0.45 nm. For the SSB site 3 also the interaction with histone H3 (depicted in pink ribbons) is displayed.

The resulting MD trajectories were first analyzed with the GROMACS clustering algorithm, using a RMSD cutoff of 0.45 nm and GROMOS method. The most representative clusters obtained for each SSB interaction site are shown in Figure 7, the Zn1 and Zn2 domains depicted in blue and red ribbons, respectively. Clearly, at each of the SSB sites 1, 2, 3 it is evident that Leu151 of Zn2 is very close to the $3^{\prime }$ terminus, while Phe44 of Zn1 remains away from the $5^{\prime }$ terminus. At each site, a number of hydrogen bonds and Van-der-Waals interactions between Zn1-Zn2 and the DNA backbone are maintained. Similar to what we observed in Ref. [18], such interactions should be responsible for stimulating the SSB gap opening, thus helping Zn2 to establish the pristine interaction with the SSB site.

In the initial analysis, we looked at the SSB gap-opening distance $d_{g}$ between the $5^{\prime }$ and $3^{\prime }$ termini flanking each SSB site, to determine whether Zn2 could stably interact with these regions. During the 200-ns MD simulation, the distance $d_{g}$ for all the three SSB sites fluctuates between 1.2 ≤ $d_{g}$ ≤ 1.6 nm, quite close to their initial separation of 1.2 nm. However, all SSBs repeatedly open the gap to larger values during the MD trajectory, up to $d_{g}$ = 2 nm, which was not observed during the 200-ns MD simulation of free SSBs without PARP1 (see Figure 6a), for which the $d_{g}$ remained at 1.4–1.6 nm. Especially for site 3 the gap has large fluctuations to $d_{g}>1.8$ nm throughout the MD simulation, in the presence of Zn1-Zn2. Altogether, it can be concluded that the presence of PARP1 brings about a substantial change in the opening distance at each SSB, to values rarely observed in the absence of Zn1-Zn2. This could seem an indication in favor of the active modification of the damage site by the enzyme (“bind–then–bend”).

**Figure 8:**
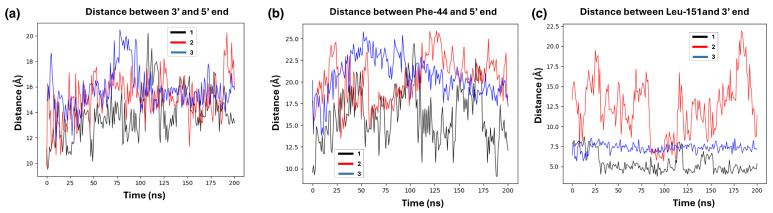
(**a**) SSB gap opening distance $d_{g}$ between the C3^′^ atoms of the $5^{\prime }$ and $3^{\prime }$ termini in the nucleosome with docked UDG. (**b**) Distance between Phe-44 and $5^{\prime }$ terminus. (**c**) Distance between Leu-151 and $3^{\prime }$ terminus. Black, red and blue lines respectively depict SSB sites 1 (middle), 2 (dyad) and 3 (end).

The next step is to understand if and how the interaction with Zn1-Zn2 can influence these changes, and to check whether the “trademark” interactions are still identified in the nucleosomal DNA, within such a limited time frame of 200 ns. As discussed in Ref. [18], the affinity of Zn2 to the $3^{\prime }$ terminus of the SSB site is notably larger than that of Zn1 towards the $5^{\prime }$ terminus. To validate this finding, we calculated the distance between Leu-151 of Zn2 and the $3^{\prime }$ terminal of the SSB sites in the simulated complex. Our results show that Leu-151 maintains close proximity to the $3^{\prime }$ end, suggesting a stronger binding affinity across all observed break sites. As illustrated in Figure 8c, the Leu151–$3^{\prime }$ distance at sites 1, and 3 fluctuates between 0.4–0.7 nm; by contrast, at site 2, Zn2 exhibits significant fluctuation in distance, ranging between 1–2 nm; however, it eventually achieves close contact around 0.5 nm after 100 ns of MD, and even shows a tendency to decrease further, at longer times. This behavior could also be attributed to a somewhat non-optimal initial docking configuration, where Zn2 was positioned a little too further away from break site, and the longer MD run could help in stabilizing the contact to the final values.

The other characteristic interaction observed in the NMR study of free DNA is the parallel stacking of Phe44 against the $5^{\prime }$ end of the DNA. As noted in our previous study of the DNA hairpin [18], this alignment is achievable only when there is a substantial opening between the two DNA SSB termini, allowing Zn2 to establish the first contact with the break site. This contact subsequently facilitates the subsequent Zn1 interaction with the $5^{\prime }$ terminus. In the case of a too narrow gap opening, which may be more typical in the more constrained conditions of the in vivo chromatin, these specific interactions are more challenging to observe. Hence, just like our previous study, we found it more difficult to bring up these interactions with the damage sites in the nucleosome as well. As depicted in Figure 8b, all the SSB sites experience a quick movement away of Zn1 within the initial few ns of the MD simulation; even though at SSB site 1 the Zn1-$5^{\prime }$ distance comes down to 1 nm, this does not appear as a stable interaction. These findings support the hypothesis that a very effective Zn1 interaction with the $5^{\prime }$ end requires a larger gap opening between the DNA termini, with Zn2 positioned closely to the $3^{\prime }$ end. This also suggests that, while Zn2 and Zn1 can individually and independently interact with SSB regions, the stability and accessibility of these interactions are influenced by site-specific fluctuations and structural adjustments, which may vary within the context of the nucleosome compared to the free DNA of the inter–nucleosome linker segments.

In addition to the role of Leu151 and Phe44 in their contacts with the $3^{\prime }$ and $5^{\prime }$ termini of the SSB just described, numerous hydrogen bonds are observed between Zn1-Zn2 and/or histone tails, and the DNA backbone at the SSB site. Significantly, Arg18 and Arg122 within the Zn1-Zn2 loop form stable H-bonds with the DNA backbone for site 1, and Arg18 alone for site 2. This same effect was observed in Ref. [18] to play a key role in inducing the bending of the hairpin–DNA structure, and is also observed in the experiments [18,33]. However, in the context of the nucleosome, and particularly at the more flexible site 3, we could not observe a similar role of Arg18 and Arg122, likely because of the more strict mechanical/structural constraints arising in the nucleosome: for this “entry” site of the NCP, the Arg122 loop should find a binding position a few nucleotides away from the $3^{\prime }$ end but the abrupt termination of the nucleosome DNA (missing an extended linker) makes such a binding position unavailable. It is therefore possible that such an interaction could be more stable in the extended nucleosome. On the other hand, Arg18 of the Zn1 loop moves away from the DNA backbone during the simulations. However, there are numerous other H-bonds made by Zn1-Zn2 either with histones, or with the DNA backbone, which help the interaction and its stronger affinity towards the break.

### 4.3. Nucleosome Reorganization by PARP1 and the Role of Histone Tails

**Figure 9:**
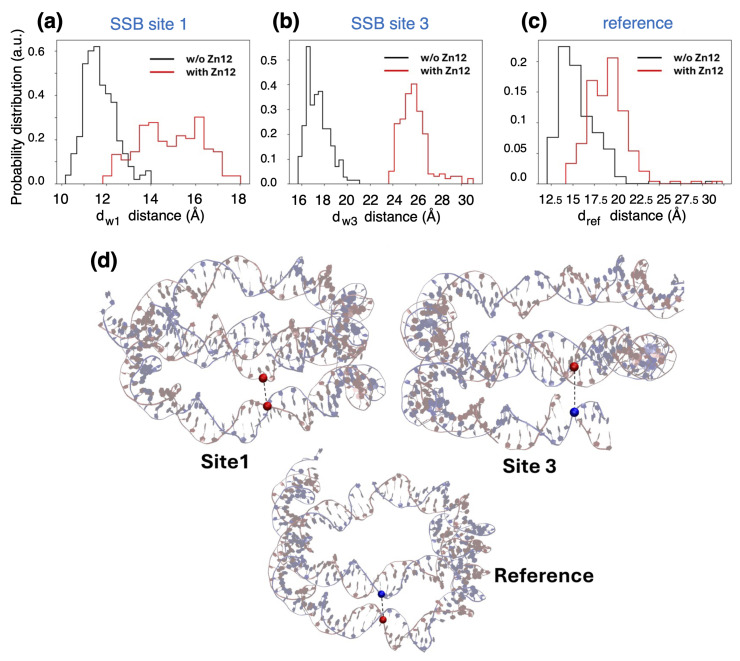
(**a**,**b**) Mechanical deformation calculated as the distance $d_{w}$ between the upper and the lower windings of nucleosome DNA at SSB sites 1,3, and (**c**) at the “reference”, unperturbed site. (**d**) Definition of $d_{w}$ for the three configurations, as the distance between the pairs of colored spheres along the phosphate backbone.

PARP1 attachment to the nucleosomal DNA including a strand-break can bring about reorganization, as well as unfolding of the nucleosome structure. Experimental studies by Maluchenko et.al. [31] performed on isolated nucleosomes, showed that interaction of the free DNA termini with two or more PARP1 copies can bring about a complete reorganization of the nucleosome, while sub-stochiometric PARP1 binding can at most bring about local changes in the DNA. Also, smFRET studies [30] showed that PARP1 attached at the linker DNA can unfold the nucleosome, as well as induce mechanical deformation, thereby helping DSB identification (however, note again that a “DSB” in such studies is represented just by the blunt ends of the 147-bp DNA in the NCP, the nucleosome core particle, and not as a true bond break on opposite strands). Similarly, in the present work including multiple SSB in the nucleosome bound to Zn-finger domains, we observe different degrees of mechanical deformation and nucleosome reorganization. As a quantitative measure of the DNA deformation, we introduce the “vertical” distance $d_{w}$ between the P atom in the unpaired nucleotide in each of SSB site 1 and 3, and the P of the closest nucleotide in the DNA winding just adjacent to it (see Figure 9d). In practice, this is a measure of the vertical distance between the two loops of DNA going around the NCP, a phenomenon sometimes dubbed as “gaping” [62]. For the sake of comparison, a similar quantity was measured between a pair of P atoms at two nucleotides in a SHL position faraway from any SSB, marked as “reference” site in Figure 9d.

Figure 9a–c plots the probability distribution of the distances $d_{w1}$, $d_{w2}$ and $d_{ref}$, between the SSB site and the DNA winding just above/below, with and without PARP1 (respectively, red and black histograms). It can be observed that there is a huge difference in the distribution, when Zn1-Zn2 is bound to the nucleosome w/r to when it is not there. For instance, the distribution of $d_{w1}$ at site 1 without Zn1-Zn2 is narrow and centered at 1.15 nm, while with the Zn1-Zn2 attached we have a much broader distribution, bimodal with maxima at $d_{w1}$ about 1.4–1.6 nm. A similar drastic change is observed for the SSB at site 3, in which case the distribution is narrow and centered at ∼1.6–1.7 nm without Zn1-Zn2, while it becomes centered at 2.1 nm with Zn1-Zn2 bound, the width of the histogram extending up to $d_{w1}≳3$ nm. (Of course, we did not measure $d_{w}$ at site 2 due to its location at the dyad, which has no secondary DNA winding above/below). To make sure that Zn1-Zn2 is actually responsible for such a mechanical distortion or deformation of the nucleosome structure, we measured the same quantity at the reference unperturbed site, which is located opposite to the dyad between SHL−3 and SHL−4, and therefore quite far from either one of the PARP1 copies. Interestingly, the results for the $d_{ref}$ reference distance display nearly similar values with and without Zn1-Zn2 docked, where both distributions fluctuate between 1.2–2.2, and 1.3–2.3 nm, respectively, only occasionally visiting larger values. Hence, these results constitute a first proof that the interaction with PARP1 can bring about partial opening as well as structural deformation of the nucleosome. Maluchenko et al. [31] observed that the distance between adjacent DNA windings could be increased up to 3.2 nm with the presence of the entire PARP1 dimer interacting with the nucleosome DNA ends (that is, at SHL–/+7). In our case, it is interesting to note that just the presence of the partial Zn1-Zn2 domains (and not the complete PARP1, that is about 20% of the total protein body) is already capable to move the two nucleosomal DNA loops away for more than 1-nm difference, and moreover well inside the NCP structure, instead of just at the DNA ends. Even though there is no complete opening of the nucleosome, it is notable that just a single Zn1-Zn2 domain was sufficient to bring about enough local deformation in the nucleosome, similar to what reported in [31].

**Figure 10:**
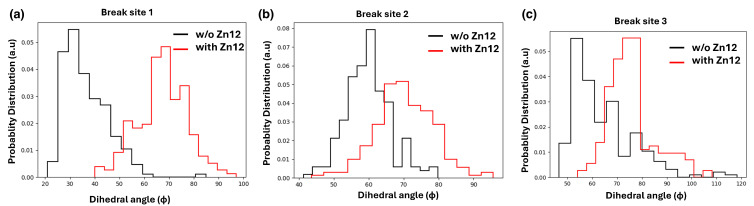
Probability distributions of the dihedral angle Φ for each break site, calculated during 200-ns MD runs with and without Zn1-Zn2 bound.

The local twisting is measured all along the MD trajectory by the dihedral angle Φ, which highlights the under/over twisting of the nucleotides flanking either side of the unpaired nucleotide. Indeed, given the large opening deformation, a significant change in the local twist values could also be expected, with vs. without Zn1-Zn2. However, the variations between the two conditions displayed in the plots of Figure 10, are in this case more subtle. The SSB site 1 displays the largest variation, with the probability distribution of Φ going from a skewed gaussian centered at $\Phi ∼30^{\circ }$ without Zn1-Zn2, to a broader gaussian shape centered at a larger over-twisting of $\Phi ∼70^{\circ }$ when Zn1-Zn2 is interacting at the SSB; SSB site 3 has a similar behavior, however with a smaller variation between the maxima, from $∼55^{\circ }$ to $∼75^{\circ }$, and a similar shape evolution of the Φ probability distribution. At the SSB dyad site 2, the difference in Φ twist angle with vs. without Zn1-Zn2 is yet less evident, the two symmetric distributions going just from about 60 to about 70° peak value. Overall, these results confirm that the presence of Zn1-Zn2 brings about a local conformational change in the nucleosome, which might help in damage identification by PARP1 auto-modification [30].

At SSB site 1 and site 3 we observed a gradual mechanical deformation developing during the MD, along the nucleotides near the $3^{\prime }$ strand, which was absent at site 2. To investigate this further, we analyzed the structural arrangement and energetics of the ensemble of 12–15 nucleotides surrounding the SSB + the Zn1-Zn2, and histones H2A, H3 and H4, using the PDBePISA 1.52 software. This analysis provided detailed information about hydrogen bonds, salt bridges, active surface area, and solvation energy (ΔG). The results revealed numerous hydrogen bonds between the DNA and proteins, with a ΔG of 1.8 kcal/mol at site 1 and —14.3 kcal/mol at site 3 for the Zn1-Zn2-Histone interaction. It is also worth noting that histone H2A makes H-bonds with a stretch of nucleotides opposite to the SSB, whereas, the N-tail of H3 makes H-bonds with the Zn1 finger, and the H4 ‘bridges’ Zn2 with the DNA $5^{\prime }$-SSB terminus.

## 5. Conclusions

In this work we studied the interaction of two different repair proteins with DNA damage in the nucleosome by an extensive set of combined docking and molecular dynamics simulations. We chose the two enzymes UDG and PARP1 occurring in the BER/SSBR repair chain of DNA damage, both being the very first actors in the identification of the respective damage site: UDG intervenes to recognize and excise a wrongly incorporated uracil in the sequence, whereas PARP1 has the role of identifying the presence of a strand break. The comparative study of how these two factors sense the key features of DNA damage in the highly complex environment of the nucleosome may be helpful in establishing similarities and differences. Our results should translate equally into an in vivo setting and are therefore representative for the processes studied. It is however clear that additional effects that we have not included into the study for reasons of feasibility - like histone tail modifications, the location of nucleosomes in chromatin arrays, ATP-dependent remodelers, or full-length PARP1 instead of the Zn1-Zn2 fragment - would render a direct observation complex, since such processes can co-occur in vivo. For example, nucleosome reorganization initiated by DNA damage sensor proteins like PARP1 through its catalytic activation, is known to promote recruitment of chromatin remodelers such as ALC1 to the lesion site [63]. These remodelers can reposition or evict nucleosomes, further enhancing lesion accessibility. Together, these in vivo mechanisms may help overcome the steric barriers posed by nucleosomal DNA, to ensure efficient repair.

The results about UDG point out some interesting, general questions about the microscopic mechanisms of damage identification by glycosylases. Firstly, it appears that only in a restricted number of situations an enzyme like UDG can find a correct arrangement on the DNA backbone, in order to form the search-complex and subsequently to move to the interrogation-complex. If we take the examples shown in Section 3.3 as a representative sample of the different positions around the nucleosome, it turns out that only ∼25% of the bases around the nucleosome are readily accessible, while the rest is either inaccessible, or perturbed by the nucleosome structure, histone tails etc. Given that on average 75% of the genomic DNA is wrapped into nucleosomes, it would mean that less than 20% of the nucleosomal DNA is amenable to direct damage repair. Secondly, it appears that, even starting from a rather correct search-complex, however minor variances in the subsequent interrogation-complex induced by structural constraints can severely hamper the next chemical step leading to the excision-complex. Third, and maybe more important, the ’hedgehog’-like nucleosome structure appears to make it difficult to imagine an enzyme like UDG freely sliding around the DNA backbone, while rapidly searching tens of adjacent bases one after another, as it has been assumed on the basis of studies on isolated DNA fragments [12,55,64]; enzyme sliding could possibly occur just on a very short range (1–2 bases next to the first one); instead, hopping between distant sites on a same nucleosome, or on a nearby one, should be the main search mechanism [65,66]; the MD simulations of direct docking to a spontaneously-flipped and pre-bent nucleotide in Section 3.3 also support this view (that is closer to a “bend-then-bind” mechanism).

As far as PARP1 is concerned, we found that it interacts with SSB sites nucleosomal DNA in a way similar to what we observed in free DNA. However, PARP1 also reorganizes the local DNA and nucleosome structure, aligning with experimental results that show its importance in modifying the chromatin structure, and histone chaperone activity during damage repair [58,67,68]. These studies show that PARP1 bound either to an isolated (mono)nucleosome on its linker DNA, or at a tri-nucleosome aggregate with free linker DNA, can reorganize and compact their structure. Even though several experimental studies report a much reduced affinity of PARP1 to nucleosomes without the DNA linker (that makes it practically undamaged nucleosomal DNA), it remains open the question about how the identification of damage could happen, when the DNA damage (notably, SSB or DSB) is located within the nucleosome structure, that is, someplace on the DNA wrapped around the histone core. The results revealed that SSBs induce local opening of nucleosomal DNA, with fluctuations in gap distance driven by both thermal and mechanical effects. The presence of Zn1-Zn2 enhanced the SSB opening, facilitating critical interactions such as the proximity of Leu-151 to the $3^{\prime }$ end, and hydrogen bonding with the DNA backbone. Compared to the same interactions observed in the experimental studies on free DNA hairpins, achieving the PARP1 “correct/trademark” interactions in the context of the nucleosome poses substantial difficulties. Also, there is no experimental literature that could support that Zn1-Zn2 interacting with nucleosome can bring about the same interactions as observed in DNA oligomer experiments. Most studies support the bending propensity of the free DNA/linker DNA, and the higher affinity of Zn1-Zn2 [30,33]. The bending propensity is out of scope for our study because, we used a mononucleosome with no free linker extending near the entry/exit points. However, we verified the high affinity of Zn2 for the $3^{\prime }$ SSB ends, and that interaction with Zn1-Zn2 can indeed bring about an increase in the gap opening at the SSB site, helping it to interact with Zn2 domain. Despite the difficulty to observe the “trademark” interactions in our model system, still there were numerous H-bonds implicating Zn1-Zn2, the histone tails and the DNA backbone, which sufficiently brought the SSB to open up, and constitute an integral part of SSB identification by PARP1.

Along with many intricate interactions, the presence of Zn1-Zn2 brought about structural reorganization in the nucleosome, by increasing the splitting between adjacent DNA gyres near the strand-break site. Altogether, these results highlight the unique structural contributions of Zn1-Zn2 to nucleosomal remodeling, underscoring its relevance as a potential focal point for understanding the PARP1 broader roles in genome stability and damage response pathways. Multiple DNA repair proteins, including DNA glycosylases, DNA polymerase-beta, APE1, are known to introduce mechanical deformation of non-nucleosomal DNA during damage recognition. Even though no direct studies showed that nucleosome reorganization directly helps recruiting the repair proteins, it has been shown at least that catalytic activation of PARP1 and its local concentration can influence H1 histone binding, which could help in recruiting repair proteins/chromatin remodelers at the damage site [30,31]. Recent observations about APE1 adopting a similar conformation during AP site recognition, both in nucleosomal DNA and non-nucleosomal DNA [69], indicate that a variable degree of nucleosome reorganization is generally needed for efficient repair. Our findings about the action of PARP1 give support to the suggestion that a DNA sculpting mechanism, similar to that observed for APE1, would explain a position-dependent enzymatic activity in the nucleosome.

Some more general conclusions can be drawn from this comparative study. Given the severe mechanical and entropic constraints of DNA in the chromatin, twisted and wrapped around a tight sequence of nucleosomes, that damage may become accessible by nucleosome ’breathing’, sliding, or DNA reptation [70,71], seems unlikely. Instead, ATP-consuming nucleosome remodeling enzymes may be more effective in lowering the energy barriers that limit spontaneous nucleosome movements (e.g., [72]). The overall scarce accessibility of the nucleosome with its complex structure, represents a dynamical obstacle to diffusion processes by which enzymes are supposed to accelerate the identification of damage by searching along the DNA: in the typical case, only about one base out of four in the nucleosome is geometrically exposed, a fraction further reduced by pertubations from histone tails. However, all these points leave the main question unanswered: how enzymes can efficiently identify DNA damage? Here and in a series of previous works [16,17,18], we pointed out that structural deformation at the defect sites, likely more severe in the case of DNA breaks while more subtle in the case of base damage, could be a supporting mechanism for damage sensing. The variable degree of deformation of the molecular structure is able to induce a stress field that propagates along the DNA (see for example our molecular stress calculations around kinked and twisted DNA strands [16,17]); for example, in the case of glycosylase damage identification, DNA kinking and twisting can increase the rate of spontaneous nucleotide flipping. Also, recent experimental studies [24] are beginning to demonstrate that DNA dynamics and mechanical deformation are indeed cooperative mechanisms in damage sensing. Such studies also highlight the possible sequence-dependence of the repair process efficiency, given the rather large variations in DNA flexibility according to the base-pairing and -stacking sequence.

## Figures and Tables

**Table 1:** Summary of the molecular interactions of UDG with each flipped-out nucleotide (T vs. U), for the various positions around the nucleosome, averaged over the last 10 ns of each 100-ns MD trajectory. For H-bonds, salt bridges, hydrophobic interactions, and $\Delta G_{S}$, the first number is the total and the second that corresponding to the flipped nucleotide only. The number of ’*’ under the tetramer sequence gives a rough estimate of its local flexibility (see text).

SHL Position	5^′^-Tetramer	3^′^-Tetramer	Histone Tail	Accessibility	Chain-Nucl.	Substitution	H-Bonds	Hydrophob.	Salt Bridges	$\Delta G_{S}$ (kcal/mol)	Interface Area (Å^2^)
+1.5	TTTT	ACCG	yes	hard	I–15	T	1 / 1	2 / 2	0 / 0	—13.5/—1.1	706
	*****	**				U	2 / 2	0 / 0	0 / 0	—10.5/—1.3	783
0 (dyad)	GCTG	CCCC	close	easy	I–3	T	8 / 4	1 / 1	1 / 0	—6.4/—2.6	502
	**	****				U	4 / 4	2 / 1	3 / 1	—9.8/—3.9	725
+0.5	GTCC	CCGC	no	hard	I–5	T	0 / 0	0 / 0	0 / 0	—3.7/—1.3	495
	**	*				U	1 / 0	0 / 0	0 / 0	—5.0/—1.3	514
+6.5	TATA	ACAT	no	medium	I–64	T	3 / 2	0 / 0	1 / 0	—11.8/—2.1	632
	***	***				U	1 / 1	1 / 1	1 / 0	—12.2/—4.2	648
+4.5	CTCC	GGCA	yes	easy	I–46	T	5 / 4	2 / 2	1 / 1	—4.9/—3.1	773
	****	**				U	7 / 4	1 / 1	2 / 1	—13.7/—2.4	1061
+3	TTAC	CCCT	no	easy	I–34	T	4 / 1	0 / 0	0 / 0	—3.2/—3.8	622
	***	****				U	8 / 6	1 / 1	0 / 0	—7.1/—3.1	687
—5	GGCT	CGGC	no	medium	J–53	T	9 / 5	3 / 1	1 / 0	—10.1/—4.8	649
	**	*				U	3 / 2	1 / 1	2 / 1	—11.2/—4.1	751

## Data Availability

Partial trajectories in GROMACS format and PDB files from the molecular dynamics simulations in this work are available at the repository FigShare, accession link: https://doi.org/10.6084/m9.figshare.28692335.v1. Additional information available from the corresponding author: fabrizio.cleri@univ-lille.fr.

## Abbreviations

The following abbreviations are used in this manuscript:

DNA	deoxyribonucleic acid
SSB	single-strand break
DSB	double-strand break
NCP	nucleosome core particle
MD	molecular dynamics
UDG	uracil DNA glycosylase
PARP	poly-ADP-ribose polymerase
DDR	DNA damage repair
BER	base-excision repair
SSBR	single-strand break repair
SHL	superhelical symmetry axis
PDB	protein data bank
RMSD	root-mean-squared displacement
RMSF	root-mean-squared fluctuation
FRET	Forster resonance energy-transfer
NMR	nuclear magnetic resonance
